# ALKBH5 in development: decoding the multifaceted roles of m^6^A demethylation in biological processes

**DOI:** 10.3389/fmolb.2025.1599487

**Published:** 2025-08-04

**Authors:** Xinye Zhang, Linfang Zhou, Cheng Tian, Huangheng Tao

**Affiliations:** ^1^ State Key Laboratory of Oral & Maxillofacial Reconstruction and Regeneration, Key Laboratory of Oral Biomedicine Ministry of Education, Hubei Key Laboratory of Stomatology, School & Hospital of Stomatology, Wuhan University, Wuhan, Hubei, China; ^2^ Xiamen Key Laboratory of Stomatological Disease Diagnosis and Treatment, Stomatological Hospital of Xiamen Medical College, Xiamen, China; ^3^ Guangdong Provincial Key Laboratory of Stomatology, Guanghua School of Stomatology, Hospital of Stomatology, Sun Yat-sen University, Guangdong, China; ^4^ Center for Cariology, Endodontics and Periodontics, School & Hospital of Stomatology, Wuhan University, Wuhan, Hubei, China

**Keywords:** ALKBH5, m^6^A demethylation, development, organogenesis, therapeutics

## Abstract

N^6^-methyladenosine (m^6^A), an abundant internal RNA modification in eukaryotes, serves as a dynamic post-transcriptional regulator of gene expression by influencing RNA splicing, stability, translation, and decay. This reversible epitranscriptomic mechanism, which is mediated by methyltransferase (writers), demethylase (erasers), and m^6^A-binding proteins (readers), is pivotal in diverse biological contexts. Among m^6^A erasers, alkylation repair homolog protein 5 (ALKBH5), an Fe(II)/α-ketoglutarate-dependent dioxygenase, is the second to be discovered and one of the most significant demethylases. Mounting evidence underscores ALKBH5’s role in modulating developmental programming, where it coordinates processes such as lineage specification, organogenesis, and tissue homeostasis. This review systematically deciphers the multifaceted contributions of ALKBH5-mediated m^6^A demethylation to developmental biology. We synthesize recent advances elucidating how ALKBH5-driven m^6^A erasure dynamically regulates transcriptomic rewiring during embryogenesis, reproductive development, cardiac development, central nervous system development, immune system development, pancreatic organogenesis, osteogenic/odontogenic differentiation, adipogenesis, and angiogenesis. These revelations not only deepen our understanding of epitranscriptomic regulation in ontogeny but also illuminate therapeutic avenues for developmental anomalies and regenerative medicine.

## 1 Introduction

Development is an incredibly intricate and elaborate process through which a single fertilized cell undergoes a series of remarkable transformations and eventually evolves into a highly complex multicellular organism (Shestopalov and Chen, 2008; Loseva and Gladyshev, 2024). Development is fundamental for life, guaranteeing survival, reproduction, and the continuity of species. This meticulously orchestrated journey encompasses cellular differentiation, tissue patterning, and organogenesis, driven by precise spatiotemporal regulation of gene expression (Warmflash et al., 2014; Mittnenzweig et al., 2021; Ohta and Yamada, 2023). An indispensable factor in development is epigenetics, which regulates gene expression without modifying the underlying DNA sequence (Bird, 2007; Taby and Issa, 2010). Key epigenetic processes, including DNA methylation, posttranslational modifications, and RNA-based mechanisms such as N^6^-methyladenosine (m^6^A) methylation, enable cells to interpret genetic information in a context-dependent manner (Matouk and Marsden, 2008). Disruptions in epigenetic regulation are linked to developmental disorders, aging, and cancer, underscoring their dual role as guardians of normal development and mediators of disease (Taby and Issa, 2010; Ashapkin et al., 2023; Wang et al., 2022). Consequently, understanding the epigenetic networks in development may contribute to unveil new frontiers in developmental biology and regenerative medicine.

The epitranscriptome, encompassing post-transcriptional chemical modifications of RNA, constitutes a fundamental regulatory layer in gene expression (Li C. et al., 2025). Among these modifications, m^6^A is the most abundant and dynamic internal modification, notably present in different RNA types including messenger RNAs (mRNAs), circular RNAs (circRNAs), micro RNAs (miRNAs), and long non-coding RNAs (lncRNAs) (Desrosiers et al., 1974; Xiao et al., 2023; Jiang et al., 2021). The deposition, removal, and recognition of m^6^A, which are respectively orchestrated by writer, eraser, and reader proteins, govern RNA metabolism at multiple levels, including splicing, stability, translation, and subcellular localization (Jiang et al., 2021; Sendinc and Shi, 2023; Zaccara et al., 2019). This reversible modification system is capable of responding to developmental cues and environmental stimuli, positioning m^6^A as a key regulator of cellular differentiation, tissue patterning and organismal development (Geula et al., 2015; Zheng et al., 2020). Among the enzymes responsible for m^6^A erasure, alkylation repair homolog protein 5 (ALKBH5), an Fe(II)/α-ketoglutarate-dependent dioxygenase, has garnered significant attention for its unique ability to selectively demethylate m^6^A in RNA species, including mRNA, circRNA, and lncRNA (Aik et al., 2014; Shao et al., 2023; Cai et al., 2024). Mounting evidence now implicates ALKBH5 as a key epigenetic regulator of development, where its demethylase activity influences embryogenesis, organogenesis, and tissue regeneration (Liang et al., 2024; Dong et al., 2023; Ma et al., 2022; Han et al., 2021a). However, there is no systematic article that comprehensively summarizes the role and regulatory mechanism of ALKBH5 in development.

This review synthesizes the current knowledge regarding the regulatory contributions of ALKBH5 to developmental biology, emphasizing its mechanistic interplay with m^6^A-modified transcripts. We first embark on a detailed description of the m^6^A modification, delving into its various characteristics and implications. Building upon this framework, we delineate the structural basis of ALKBH5’s enzymatic activity and substrate recognition, providing a molecular framework for its developmental functions. Subsequently, we dissect its subtle yet significant stage-specific influences in certain development processes such as embryogenesis, neurodevelopment, reproductive biology, and organogenesis, focusing on its regulation of key mRNA or signaling pathways (e.g., Wnt/β-catenin, PI3K/AKT) through selective m^6^A erasure. Finally, we briefly introduced the therapeutic strategies specifically targeting ALKBH5 and discussed its potential in treating developmental disorders.

## 2 m^6^A modifications: a dynamic regulatory layer in RNA biology

### 2.1 m^6^A modifications

RNA maturation requires a wide variety of enzymes for its chemical modification. To date, over 170 types of chemical modifications have been identified on RNA (Wang M. K. et al., 2023). Among them, m^6^A, which was first discovered in 1974 and is the most abundant internal chemical modification in eukaryotic mRNA, accounting for over 80% of all RNA methylation modifications, has emerged as a pivotal post-transcriptional regulator of gene expression (Desrosiers et al., 1974; Xiao et al., 2023; Zheng et al., 2020).

**FIGURE 1:**
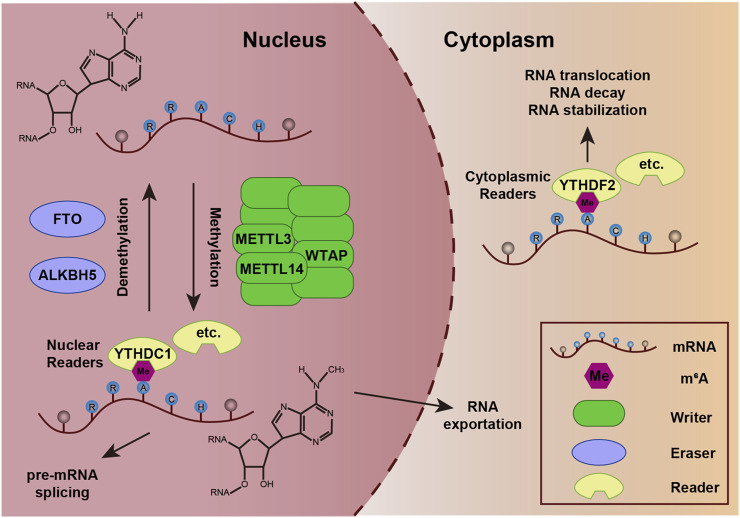
Dynamic regulation of m^6^A modification on mRNA. Methyltransferase complexes (e.g., METTL3-METTL14-WTAP) catalyze the addition of methyl groups (CH_3_) to adenine residues at conserved motifs (e.g., RRACH) on mRNA. Demethylases (FTO and ALKBH5) remove methyl groups, reversing m^6^A modification and enabling dynamic regulation of mRNA fate. m^6^A-binding proteins (YTHDC1, YTHDF2, etc.) recognize and bind to m^6^A sites, mediating downstream effects such as mRNA splicing, nuclear export, stability, degradation, or translation.

This reversible modification is dynamically deposited by methyltransferases (writers) and demethylases (erasers), and is recognized by specific binding proteins (readers) (Jiang et al., 2021; Sendinc and Shi, 2023). The methyltransferase complex, primarily comprising METTL3, METTL14, and WTAP, can install methyl groups on adenosine residues within consensus sequences (e.g., RRACH) to catalyze m^6^A modification (Xu and Ge, 2022). Conversely, demethylases, such as FTO and ALKBH5, mediate its removal, ensuring dynamic regulation (Gao et al., 2024). The “readers” including YTHDC and YTHDF families, decode m^6^A signals by binding to modified RNAs, thereby directing their fate (Yen and Chen, 2021). The m^6^A process governs diverse aspects of RNA metabolism, exerting a significant influence on a wide range of cellular processes, ranging from development to disease (Figure 1).

### 2.2 Biological functions of m^6^A

m^6^A encompasses roles in development, immune modulation, and disease pathogenesis. As a versatile modulator of RNA fate, m^6^A is significant in tuning transcript half-lives, guiding spliceosome assembly, licensing nuclear export, and reprogramming translation.

m^6^A dynamically controls mRNA stability through context-dependent interactions with reader proteins. For example, m^6^A can recruit YTHDF2 to participate in and promote the process of mRNA decay actively (Li et al., 2018; Zaccara and Jaffrey, 2020). *YTHDF1* deletion prolongs the half-lives of mRNAs, thereby causing a degradation delay of mRNAs (Zaccara and Jaffrey, 2020; Li et al., 2022). m^6^A precisely fine-tunes alternative splicing events, playing a crucial role in regulating the diversity and complexity of gene expression. For instance, the reader YTHDC1 can recruit and modulate splicing factors to facilitate their access to the binding regions of targeted mRNAs (Xiao et al., 2016). m^6^A modification also impacts the RNA nuclear export. YTHDF3 has been proposed to act as an mRNA-transferring protein (Zaccara and Jaffrey, 2024). Additionally, YTHDC1 facilitates m^6^A-marked mRNA export from the nucleus, ensuring timely cytoplasmic translation (Roundtree et al., 2017). Furthermore, m^6^A plays a crucial role in regulating translation efficiency. 5′UTRs m^6^A can recruit eukaryotic initiation factor 3 to initiate cap-independent translation under stress conditions (Meyer et al., 2015). YTHDF1 can promote the translation of m^6^A-mRNAs (Zaccara and Jaffrey, 2024; Zou et al., 2023). In addition to the aforesaid functions, m^6^A extensively regulates non-coding RNAs (e.g., lncRNAs, miRNAs) to broaden its functional range (Huang et al., 2020).

## 3 ALKBH5: a key RNA demethylase in epitranscriptomic regulation

ALKBH5 is the second discovered demethylase and was first reported as a mammalian demethylase in 2013 (Zheng et al., 2013a; Zheng et al., 2013b). It belongs to the ALKB family of Fe(II)/α-ketoglutarate-dependent dioxygenases. Specifically, it plays a crucial role in catalyzing the oxidative demethylation of m^6^A on RNA substrates without generating any intermediate products (Shen et al., 2014).

**FIGURE 2:**
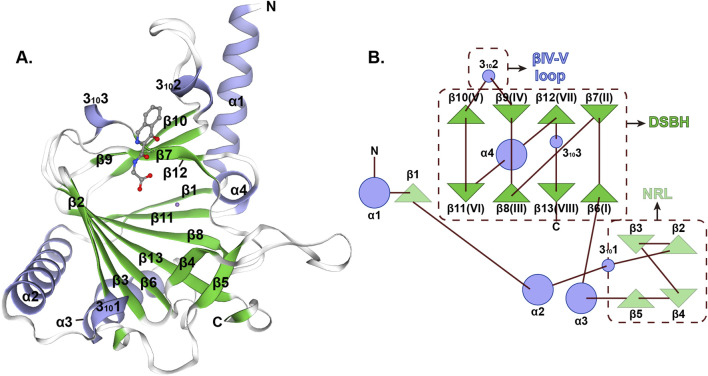
Structural characterization of human ALKBH5 protein **(A)** Overall three-dimensional structure of ALKBH5 (PDB ID: 4NJ4). **(B)** Topology of the structure of ALKBH5_66–292_.

The three-dimensional architecture of ALKBH5 exhibits a sophisticated organization featuring multiple α-helices, β-strands, and random coil that demonstrate precise spatial coordination (Figure 2A) (Aik et al., 2014). The active center of ALKBH5 is a highly conserved catalytic pocket known as the double-stranded β-helix (DSBH), which can coordinate Fe(II) and α-ketoglutarate (2-oxoglutarate, 2OG) to catalyze the oxidative demethylation of m^6^A in RNA (Qu et al., 2022; You et al., 2022). This remarkable structure has two distinct β-sheets: the major β-sheet, which is composed of strands β6, 8, 11, and 13, and the minor β-sheet, which consists of strands β7, 9, 10, and 12 (Figure 2B). The space between the two β-sheets serves as a passage, facilitating the substrate’s access to the active site, thereby enabling the efficient execution of the catalytic process. A loop extending from the DSBH is located between the β9 and β10 strands, conferring single-stranded RNA selectivity (Figure 2B) (Aik et al., 2014). Additionally, ALKBH5 contains a nucleotide recognition lid (NRL), which is a flexible loop region adjacent to its catalytic core and possesses β2, 3, 4, and 5 (Figure 2B). NRL is capable of interacting with m^6^A on RNA, ensuring precise positioning of the target nucleotide within the catalytic pocket, which plays a pivotal role in substrate binding (Qu et al., 2022; Feng et al., 2014). Collectively, the DSBH provides the enzymatic framework, whereas the NRL and βIV-V loop adjust substrate recognition and binding. These domains enable ALKBH5 to selectively demethylate m^6^A on RNA, impacting processes like mRNA splicing, stability, and translation.

## 4 ALKBH5: orchestrating multifaceted developmental programs

Recent studies underscore that precise epitranscriptomic reprogramming, driven by context-dependent m^6^A demethylation, is essential for diverse developmental programs. Central to this reprogramming is the active removal of m^6^A marks by erasers, including ALKBH5, which enables rapid transcriptome rewiring in response to developmental cues. Building on this paradigm, the subsequent sections elaborate on how ALKBH5 functions in embryogenesis, reproductive development, cardiac development, central nervous system development, immune system development, pancreatic organogenesis, osteogenic differentiation, odontogenic differentiation, adipogenesis, and angiogenesis, uncovering the mechanisms for its role as a developmental modulator.

### 4.1 ALKBH5 in embryogenesis

Embryogenesis refers to the complicated developmental process by which a zygote undergoes cell proliferation, differentiation, and morphogenesis to form a structured embryo with functional organ systems (Ming et al., 2022; Du et al., 2022). A pivotal milestone in embryogenesis is gastrulation, during which the three germ layers (ectoderm, mesoderm, endoderm) are established (Lu et al., 2001). Notably, the commitment of the definitive endoderm represents a critical event in early cell fate specification (Robb et al., 2004).

ALKBH5 has been proved to possess the ability to influence the human endoderm fate (Liang et al., 2024). Knockout of *ALKBH5* disrupts definitive differentiation and primitive streak specification in human embryonic stem cells (ESCs). Mechanistically, *ALKBH5* deficiency destabilizes *GATA6* mRNA in a YTHDF2-dependent manner. On the other hand, ALKBH5 can remove m^6^A modifications from *GATA6* mRNA, enhancing its stability and translation efficiency. Then, GATA6 directly upregulates the expression of DKK1 and DKK4, which are key regulators of the Wnt/β-catenin signaling pathway, promoting the expression of endoderm-specific genes such as *SOX17* and *FOXA2* and guaranteeing the proper differentiation of human ESCs into definitive endoderm cells. At present, the doxycycline-inducible dCas13a system, when fused to the catalytic domain of ALKBH5, enables precise and reversible m^6^A demethylation at targeted mRNA sites (Chen X. et al., 2021). This engineered tool enhances mRNA stability while minimizing off-target effects, demonstrating high spatiotemporal specificity. Notably, site-specific m^6^A erasure at a single site of *SOX2* mRNA suffices to regulate the differentiation of human ESCs. Another study highlights that the circRNA Hsa_circ_0069443 can bind to ALKBH5 in trophoblast cells (Li B. X. et al., 2025). It governs the stability and expression of *FN1* through m^6^A methylation-dependent regulation, forming a functional epitranscriptomic axis essential for embryonic implantation and adhesion.

### 4.2 ALKBH5 in reproductive development

Reproductive development is a highly intricate and dynamic process initiated from the formation of the fertilized egg, encompassing the gradual formation, differentiation, and functional maturation of reproductive organs (Lochab and Extavour, 2017; Chan and Hirashima, 2022). This process plays a pivotal role in individual growth and development, as it not only dictates sexual characteristics and reproductive capacity but also profoundly influences hormonal regulation and systemic metabolic homeostasis (Du et al., 2022).

ALKBH5 plays a key role in male fertility by orchestrating spermatogenesis, while its dysregulation is linked to reproductive failure (Tang et al., 2017; Chen et al., 2022). This enzyme specifically regulates the m^6^A modification of mRNAs involved in spermatogenic processes, such as meiosis and spermatid differentiation. ALKBH5 is highly expressed in male mice testes, and its deficiency leads to abnormal spermatogenesis, reduced sperm count, impaired sperm motility, diminished testicular size, and male infertility (Zheng et al., 2013a; Tang et al., 2017; Hong et al., 2022). The underlying mechanism involves ALKBH5-mediated m^6^A demethylation of critical transcripts, particularly those spermatogenesis-related mRNAs involved in the p53 functional interaction network. This post-transcriptional regulation ensures both mRNA proper stability and efficient translation during spermatogenesis. Beyond its germ cell-autonomous functions, ALKBH5 exhibits essential roles in somatic niche maintenance. In the testicular interstitium, leydig cells (LCs) serve as the primary source of testosterone, which is a crucial hormone for male sexual development (Chen et al., 2009). ALKBH5 is upregulated during LC differentiation, where it regulates testosterone synthesis by promoting *PPM1A* translation and decreasing *CAMKK2* stability (Chen Y. et al., 2021). In Sertoli cells, it maintains blood-testis barrier integrity through m^6^A-dependent regulation of *Cdh2* mRNA translation, which is critical for basal ectoplasmic specialization dynamics (Cai et al., 2022). Additionally, ALKBH5 regulated the RNA methylation level and gene expression of *SOX9* mRNA as well as negatively regulated the proliferation of immature porcine Sertoli cells (Chen C. et al., 2023).

In oocytes, ALKBH5 regulates the m^6^A modification of maternal mRNAs, which is critical for oocyte maturation and meiotic progression. Dysregulation of ALKBH5 can lead to defects in oocyte maturation and reduced fertility (Sun et al., 2022). ALKBH5 ensures timely maternal RNA degradation during oocyte maturation by dynamically erasing m^6^A marks, thereby preventing stabilization of transcripts via the m^6^A reader IGF2BP2; loss of ALKBH5 disrupts RNA clearance through persistent m^6^A-IGF2BP2 interactions, leading to defective meiosis and female infertility (Bai et al., 2023).

### 4.3 ALKBH5 in cardiac development and regeneration

The heart is the first functional organ to develop during organogenesis, which is precisely situated in the mediastinum, occupying a position behind the sternum and between the two lungs (Marano et al., 2011; Paige et al., 2015). The heart is irreplaceable in sustaining life. It acts as a muscular pump that continuously drives oxygenated blood to all tissues via systemic circulation and deoxygenated blood to the lungs for gas exchange through pulmonary circulation (Litviňuková et al., 2020). This dual-pump mechanism ensures oxygen, nutrients, and hormones are delivered to cells while eliminating metabolic waste.

Some studies confirmed a gradual decrease in the expression of ALKBH5 in cardiac tissue after birth and emphasized the significant role of ALKBH5 in the regulation of cardiomyocytes (Han et al., 2021a; Semenovykh et al., 2022). *Alkbh5* knockout impaired cardiac regeneration and function in mice neonatal apex resection models, whereas its overexpression enhanced cardiomyocyte proliferation and restored cardiac function post-myocardial infarction (Han et al., 2021a). Mechanistically, the m^6^A modification mediated by ALKBH5 was of crucial importance as it enhanced the expression of *YTHDF1* by regulating the stability of the corresponding mRNA. This modulation ultimately facilitated the translation of *YAP*, which is recognized as a core regulator governing cardiomyocyte proliferation and the process of heart regeneration. Other research discovered that ALKBH5 is responsible for the cardiomyocyte fate determination of human ESCs from mesoderm cells and mouse pluripotent stem cells (Dong et al., 2023; Han et al., 2021b). Mechanistically, the loss function of ALKBH5 regulated the mRNA stability of *KDM5B* and *RBBP5*, which in turn promoted the expression of *GATA4* by enhancing histone H3 Lys4 trimethylation at its promoter region, thereby facilitating cardiac differentiation.

### 4.4 ALKBH5 in central nervous system development

The development of the central nervous system (CNS) refers to the process by which the brain and spinal cord form and mature from the early stages of embryonic development through childhood and adolescence (Yang et al., 2025). It involves a series of complex events such as neural tube formation, neuronal migration, axon guidance, synapse formation, and myelination. The proper development of the CNS is of utmost importance for an individual’s physical and mental wellbeing. It is the foundation for all cognitive functions including learning, memory, perception, and decision-making (Rice and Barone, 2000).

The development of the CNS requires precise spatiotemporal regulation of gene expression, with RNA methylation dynamics emerging as an important regulatory layer. The ALKBH5 protein exhibits widespread expression across brain regions, with predominant localization in neurons (Du et al., 2020). Its expression displays a dynamic developmental pattern: it is highly abundant during embryonic stages of brain development but declines progressively in late stages. Disrupted m^6^A methylation patterns can lead to developmental delays and functional abnormalities in the cerebellum. For instance, knockout of *Alkbh5* under hypoxic conditions results in disordered m^6^A levels in a subset of cell fate determination genes (such as *Cenpe*, *Cdca2*, *Ddx11*, and *Notch3*), accelerated RNA nuclear export, causes abnormal cell proliferation and differentiation in the cerebellum, and significant cerebellar development delays (Ma et al., 2018). Notably, the cerebellar integrity preserved by ALKBH5 extends to aging populations (Fei et al., 2023). Additionally, ALKBH5 may be a potential target for promoting axon regeneration in both CNS and peripheral nervous systems. The study by Wang et al. demonstrated that *Alkbh5* knockdown increased retinal ganglion cell survival rates and the number of regenerated axons (Zheng et al., 2023). The mechanism underlying this effect involves the regulation of lipid metabolism through the demethylation of *Lpin2* mRNA. *Alkbh5* knockdown reduces *Lpin2* mRNA stability by increasing m^6^A modification on its 3′UTR, thereby enhancing axon regeneration. Concisely, ALKBH5 plays a key role in CNS development and function, regulating key processes such as cerebellar development, neuronal survival, and axonal regeneration.

### 4.5 ALKBH5 in immune system development

Proper immune system development is crucial for establishing immune competence, as it involves the maturation of immune cells and the establishment of immune tolerance, which together determine the body’s capacity to respond effectively to infections and prevent autoimmune diseases (Simon et al., 2015). T cells are essential for adaptive immunity, and their maturation in the thymus involves complex regulatory mechanisms (Thapa and Farber, 2019). Based on the expression of αβ and γδ receptors, T cells are mainly divided into αβ and γδ T cells.

ALKBH5 serves as an important regulator in T cell development, particularly influencing the differentiation and expansion of γδ T cells (Zhao et al., 2023). Specifically, *Alkbh5* deficiency leads to a significant expansion of γδ T cells through enhanced proliferation and developmental programming, ultimately improving host defense against gastrointestinal *Salmonella typhimurium* infection, rather than affecting αβ T cells homeostasis (Zhao et al., 2023; Ding et al., 2022). The molecular mechanism involves m^6^A RNA modification dynamics: *Alkbh5* deficiency elevates m^6^A levels, triggering specific mRNA degradation of key Notch signaling components including *Jagged1* and *Notch2*. This mechanism elucidates the checkpoint function of m^6^A modification in T cell lineage commitment and unveils potential therapeutic targets for modulating γδ T cell-driven immune responses.

### 4.6 ALKBH5 in pancreatic organogenesis

The pancreas, an organ derived from the endoderm, is situated posterior to the stomach, with its head ensconced in the duodenal loop and its tail extending towards the spleen (Edlund, 2002). It has dual functions (the exocrine function and the endocrine function), which play a crucial role in glucose homeostasis and nutrient digestion (Alonge et al., 2023; Larsen and Grapin-Botton, 2017). In recent years, numerous transcription factors, such as MNX1, PDX1, NKX6.1, and SOX9, which play crucial roles in the organogenesis of the pancreas, have been identified (Cano et al., 2014).

Previous research has verified that ALKBH5 regulates pancreatic organogenesis by regulating RNA m^6^A demethylation (Ma et al., 2022). The research team discovered that ALKBH5 maintains the balance of m^6^A modifications on transcripts essential for pancreatic progenitor differentiation. Specifically, ALKBH5-mediated removal of m^6^A marks stabilizes key mRNAs encoding transcription factors like MNX1, SOX9, PDX1, and NKX6.1 to evade the YTHDF2-mediated mRNA decay pathway, thereby regulating human pancreatic differentiation. Additionally, the cofactor of ALKBH5, namely, α-ketoglutarate, could also exert functions in this organ differentiation.

### 4.7 ALKBH5 in osteogenic and odontogenic differentiation

Osteogenic differentiation refers to the fundamental biological process by which mesenchymal stem cells or osteoprogenitor cells (such as bone progenitor cells) undergo progressive maturation into functional osteoblasts under precise regulatory control (Valenti et al., 2016). As a cornerstone of skeletal development and homeostasis, this complex process involves a multifaceted cascade of biological events, including specific gene activation, coordinated signaling pathway regulation, extracellular matrix biosynthesis, and subsequent mineralization processes, ultimately culminating in the formation of functional bone tissue (Deng et al., 2008).

ALKBH5 exhibits context-dependent roles in osteogenic differentiation by dynamically regulating RNA of key osteogenic factors. For instance, ALKBH5 was upregulated during osteoblast differentiation and promotes osteogenesis by enhancing the stability of *Runx2* mRNA, a master transcription factor for osteoblast differentiation (Feng et al., 2021). Additionally, ALKBH5 dynamically reverses the METTL3-driven m^6^A modification of *MYD88* mRNA, thereby suppressing NF-κB signaling to facilitate osteogenic differentiation of mesenchymal stem cells (MSCs) (Yu et al., 2020). In the pathological context of ligamentum flavum ossification, an ectopic ossification disorder characterized by aberrant bone formation within spinal ligaments, ALKBH5 shows elevated expression and functionally drives the mineralization process in ligament flavum cells (Wang H.-F. et al., 2020). Mechanically, ALKBH5 facilitates osteogenesis by demethylating *BMP2* and activating the AKT signaling pathway. These findings highlight ALKBH5’s role as a positive regulator of osteogenesis through m^6^A-dependent modulation of transcription factors and signaling pathways. However, ALKBH5 can also exert inhibitory effects on osteogenic differentiation. In senescent bone marrow mesenchymal stromal cells, ALKBH5 suppresses osteogenic differentiation by reducing m^6^A modification on *VDAC3* mRNA and accelerating its degradation, which is a mitochondrial ROS sensor critical for counteracting cellular senescence (Huang Y. et al., 2024). Another study identified a distinct inhibitory axis where ALKBH5 destabilizes *PRMT6* mRNA and enhances its decay, suppressing PI3K/AKT pathway and osteogenic differentiation (Li et al., 2021). These contrasting roles reflect ALKBH5’s functional duality, influenced by cellular senescence status, target mRNA specificity, and downstream signaling cross-talk.

Odontogenic differentiation constitutes a specialized cellular reprogramming event wherein dental pulp stem cells transition into polarized odontoblasts, the principal secretory cells governing dentin matrix synthesis (Wu et al., 2024). This differentiation cascade serves dual physiological imperatives: (1) establishing the primary dentin architecture during tooth development, and (2) mobilizing reparative dentinogenesis in response to carious or mechanical stimulis (Huang D. et al., 2024; Tian et al., 2022).

ALKBH5 may play a role analogous to its function in osteogenic differentiation during odontogenic differentiation. Experimental evidence indicates that ALKBH5 is upregulated during odontoblast differentiation (Tian et al., 2022). Conditional deficiency of *Alkbh5* reduces odontoblast numbers and promotes pre-dentin formation, though it is important to note that the observed phenotype is not striking. Mechanistically, ALKBH5 promotes dentin matrix formation through a molecular strategy: epigenetic stabilization of *Runx2* mRNA through m^6^A demethylation, and enhancement of the PI3K/AKT signaling pathway. However, research on the relationship between ALKBH5 and odontoblastic differentiation is currently limited, and more studies are needed in the future to fully understand its role in this process.

### 4.8 ALKBH5 in adipogenesis

Adipogenesis, a highly plastic and dynamic process, drives the phenotype of functionally mature adipocytes (the defining cell type of adipose tissue) (Fève, 2005). Adipose tissue serves as a critical site for lipid storage, systemic energy homeostasis, and insulin sensitivity regulation (Sarjeant and Stephens, 2012). m^6^A methylation has been demonstrated to regulate various aspects of mRNA metabolism during adipogenesis (Wang L. et al., 2020; Song et al., 2020).

During adipogenic differentiation, ALKBH5 expression progressively declines, leading to TRAF4 downregulation through its m^6^A RNA demethylation activity (Cen et al., 2020). Mechanistically, TRAF4 forms a functional complex with PKM2 to activate β-catenin signaling, thereby establishing an anti-adipogenic regulatory axis. Consequently, the depletion of ALKBH5 can enhance adipogenesis of MSCs.

### 4.9 ALKBH5 in angiogenesis

Angiogenesis, the growth of blood vessels from existing vasculature, is integral to development (organ formation) and adaptation (tissue repair) (Chen et al., 2017). ALKBH5 is regarded as a significant regulator of angiogenesis. Nevertheless, current research primarily focuses on its roles in pathological or hypoxic conditions, while its involvement in developmental angiogenesis remains underexplored. For instance, ALKBH5 acts as a negative regulator of post-ischemic angiogenesis through post-transcriptional modulation and destabilization of *WNT5A* mRNA in an m^6^A-dependent manner (Zhao et al., 2021). Conversely, it sustains angiogenesis in endothelial cells under acute ischemic stress by reducing m^6^A methylation of *SPHK1* mRNA (Kumari et al., 2021). Furthermore, specific deletion of *Alkbh5* in the murine hematopoietic system attenuates stress-induced hematopoietic fitness through regulation of *Ogdh* mRNA stability (Gao et al., 2023).

## 5 Therapeutic targeting of ALKBH5: from molecular inhibitors to clinical applications

As a pivotal m^6^A RNA demethylase, ALKBH5 has emerged as a therapeutic target due to its dysregulation in diverse pathological conditions. Current targeting strategies encompass inhibitors, proteolysis targeting chimera, programmable m^6^A-editing systems, compounds targeting the regulatory machinery of ALKBH5, as well as gene therapy approaches (Qu et al., 2022). Among these, pharmacological inhibition represents the most straightforward therapeutic paradigm.

A variety of ALKBH5 inhibitors have been developed, including natural, clinical pharmacological, and small-molecule inhibitors (Fang et al., 2025). For example, citrate, a natural inhibitor of ALKBH5, disrupts the demethylase activity of ALKBH5 by directly binding to it and replacing Fe(II) and 2OG (Xu et al., 2014). IOX1, the clinical pharmacological inhibitors of ALKBH5, competitively inhibits 2OG binding and suppresses ALKBH5, which demonstrates protective effects against acute kidney injury and sevoflurane-induced neuronal damage in the hippocampus (Li et al., 2016; Chen J. et al., 2023; Meng et al., 2024). In addition, several new small-molecule inhibitors of ALKBH5 have been developed. The binding site of imidazobenzoxazin-5-thione MV1035 in ALKBH5 partially overlaps with that of 2OG, inhibiting the demethylation activity of ALKBH5, which suppresses migration and invasion in glioma cell lines (Malacrida et al., 2020). Novel inhibitors Ena15 and Ena2 show differential inhibition modes (non-competitive or competitive 2OG binding) with efficacy against the growth activity of glioblastoma multiforme (Gao et al., 2024; Takahashi et al., 2022). In addition to the above ALKBH5 inhibitors, there are many unlisted, such as cmp-3, cmp-6, DO-2728 and so on (Selberg et al., 2021; Wang Y.-Z. et al., 2023). These compounds effectively modulate m^6^A level in target mRNAs, establishing ALKBH5-targeted therapy as a promising strategy for various human diseases.

Despite significant advances in ALKBH5 inhibitor development for certain disorders (such as oncology), their therapeutic potential in developmental pathologies remains an under-investigated frontier. During development, m^6^A modification plays a dynamic regulatory role in critical biological processes such as embryogenesis, neurogenesis, and organogenesis. ALKBH5 potentially influences these events by altering the expression of development-related genes. However, existing studies predominantly focus on post-developmental disease contexts, leaving the mechanistic and therapeutic implications of ALKBH5 inhibitors in developmental anomalies (e.g., neural tube defects, congenital malformations) unaddressed. Future investigations should integrate developmental models to elucidate how ALKBH5-mediated m^6^A remodeling governs developmental programs and evaluate the feasibility of pharmacological inhibition to intervene in abnormal developmental processes. This paradigm shift from disease treatment to developmental pathway modulation could broaden the clinical applicability of ALKBH5 inhibitors and offer novel strategies for targeting developmental disorders.

## 6 Conclusion and discussion

**FIGURE 3:**
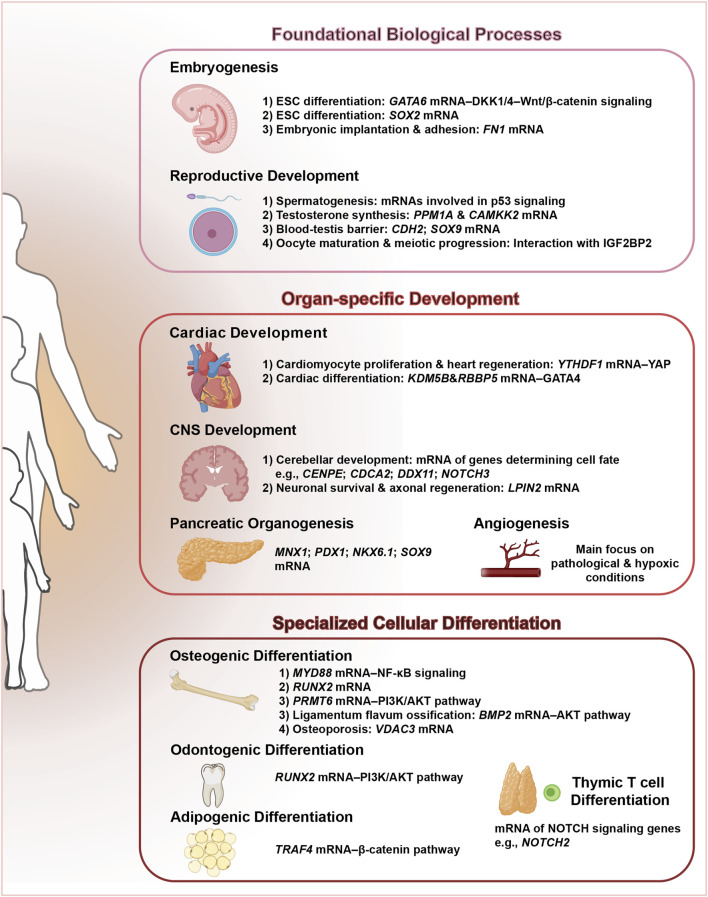
ALKBH5: a central regulator orchestrating multifaceted developmental programs. This schematic illustrates the pivotal role of ALKBH5, an m^6^A demethylase, in coordinating diverse developmental processes.

In summary, ALKBH5 has emerged as an important epigenetic regulator that intricately influences a multitude of developmental processes through the erasure of m^6^A RNA methylation (Figure 3; Table 1). This enzyme coordinates a hierarchical regulatory network across three fundamental dimensions: (1) foundational biological processes including embryogenesis and reproductive system maturation, where it maintains developmental plasticity; (2) organ-specific development spanning cardiac morphogenesis, pancreatic organogenesis, CNS development, and angiogenesis, demonstrating remarkable tissue adaptability; and (3) specialized cellular differentiation programs encompassing osteogenic differentiation, dentin matrix organization, adipogenesis, and thymic T cell differentiation. The precise regulation of RNA stability, splicing, and translation by ALKBH5-mediated m^6^A demethylation is crucial for orchestrating the complex molecular and cellular events underlying these developmental programs. The growing body of evidence underscores the significance of ALKBH5 in maintaining the integrity and functionality of various tissues and organs during development.

**TABLE 1:** The impact of ALKBH5 on development.

Development	Expression	Target RNAs	mRNA stability	Target pathways	Functions	Ref.
Embryogenesis	-	*GATA6*	Increase	Wnt/β-catenin pathway	Promote ESCs differentiation	Ashapkin et al. (2023)
	-	*SOX2*	Increase	-	Promote ectodermal differentiation of ESCs	Chen C. et al. (2021)
	-	*FN1*	Decrease	-	Inhibit trophoblast cell proliferation, migration, invasion	Li C. et al. (2025)
Reproductive Development	-	*Dnmt1* *Uhrf1*, et al.	Increase	p53 pathway	Promote spermatogenesis	Zheng et al. (2013a)
	-	*Sycp1*, *Sycp2*, et al.	Increase	-	Promote spermatogenesis	Tang et al. (2017)
	-	-	-	-	Promote spermatogenesis	Hong et al. (2022)
	Up	*PPM1A*	-	-	Promote testosterone synthesis	Chen J. et al. (2021)
		*CAMKK2*	Decrease			
	-	*Cdh2*	-	-	Promote blood-testis barrier integrity via basal endoplasmic specialization	Cai et al. (2022)
	Up	*SOX9*	Decrease		Inhibit immature porcine Sertoli cell proliferation	Chen C. et al. (2023)
Cardiac Development	Down	*YTHDF1*	Increase	-	Promote cardiomyocyte proliferation	Han et al. (2021a)
		-	-	-	-	Semenovykh et al. (2022)
		-	-	-	Inhibit the early stage of stem cell cardiac differentiation	Dong, et al. (2023)
		*KDM5B*	Increase	-	Inhibit cardiomyocyte differentiation of ESCs	Han et al. (2021b)
		*RBBP5*	Decrease			
CNS Development	Down	*Cdca2* *Ddx11* *Notch3*, et al.	Increase or Decrease	-	Inhibit cerebellar development	Ma et al. (2018)
	-	*Lpin2*	Increase	-	Inhibit axonal regeneration	Zheng et al. (2023)
Osteogenic Differentiation	Up	*Runx2*	Increase	-	Promote osteoblasts differentiation and mineralization	Feng et al. (2021)
	-	*MYD88*	Decrease	NF-κB pathway	Promote osteogenesis of MSCs	Yu et al. (2020)
	Up	*BMP2*	-	AKT pathway	Promote osteogenesis of ligamentum favum cells	Wang L. et al. (2020)
	-	*VDAC3*	Decrease	-	Inhibit osteogenic differentiation of etoposide-induced senescent cells	Huang D. et al. (2024)
	Down	*PRMT6*	Decrease	PI3K/AKT pathway	Inhibit osteogenic differentiation of MSCs	Li et al. (2021)
Odontogenic Differentiation	Up	*Runx2*	Increase	PI3K/AKT pathway	Promote odontogenic differentiation	Tian et al. (2022)
Adipogenesis	Down	*TRAF4*	-	β-catenin pathway	Inhibit adipogenesis of MSCs	Cen et al. (2020)

The therapeutic potential of targeting ALKBH5 in developmental disorders and regenerative medicine is promising. For instance, modulating ALKBH5 activity could offer strategies to enhance embryonic development, improve fertility, promote cardiac regeneration, and alleviate neuroinflammatory conditions. Moreover, understanding the specific m^6^A-modified transcripts and signaling pathways regulated by ALKBH5 in different developmental contexts may lead to the development of targeted therapies for various diseases associated with aberrant RNA methylation patterns.

However, several challenges remain: (1) ALKBH5 serves as a key regulator of mammalian development, as demonstrated by phenotypes in *Alkbh5* knockout mouse models across germline development (Zheng et al., 2013a; Tang et al., 2017), cardiac repair (Han et al., 2021a), immune system development (Ding et al., 2022), cerebellar development (Ma et al., 2018), osteogenesis (Li et al., 2021), and odontogenesis (Tian et al., 2022). Although ALKBH5 expression peaks in testes, its functional impact in other non-testicular tissues demonstrates that physiologically relevant m^6^A demethylation occurs even at lower expression levels. However, proposed roles in broader developmental contexts, such as pancreatic organogenesis and adipogenesis, primarily derive from *in vitro* or correlative evidence, lacking direct validation in *Alkbh5* knockout mice. This absence of *in vivo* confirmation suggests ALKBH5 might not be essential for these specific processes. Potential contributing factors may involve limited expression of ALKBH5 in relevant tissues, compensatory activity by related demethylases (e.g., FTO) or functional redundancy within epigenetic regulatory pathways. (2) The most commonly used method to map m^6^A and to detect changes in m^6^A is antibody-dependent techniques (e.g., MeRIP-seq/m6A-seq). In some cited studies, MeRIP-seq was performed to screen for molecules regulated by ALKBH5 (Cai et al., 2022; Tian et al., 2022). Although MeRIP-seq can indicate approximate sites of m^6^A, it can’t be used to quantitatively measure the precise fraction of transcript copies modified by m^6^A (McIntyre et al., 2020). And low-abundance m^6^A sites in critical genes may evade detection despite functional importance. Consequently, the need for further research and alternative assays (e.g., m^6^A-seq2 (Dierks et al., 2021), GLORI (Liu et al., 2023) and nanopore sequencing (Grigorev et al., 2024)) to resolve the ALKBH5-dependent changes at specific m^6^A sites. (3) The dynamic and context-dependent nature of m^6^A methylation requires a deeper understanding of the spatiotemporal regulation of ALKBH5 and its interplay with other epitranscriptomic factors. (4) Therapeutic targeting requires careful evaluation of off-target effects and interplay with epigenetic modifications. Future research should focus on elucidating the molecular mechanisms underlying ALKBH5’s functions in development, exploring its role in disease pathogenesis, and developing precise tools to modulate its activity for therapeutic benefits.

## Abbreviations

2OG, 2-oxoglutarate; ALKBH5, alkylation repair homolog protein 5; circRNAs, circular RNAs; CNS, central nervous system; DSBH, double-stranded β-helix; ESCs, embryonic stem cells; LCs, leydig cells; lncRNAs, long non-coding RNAs; m^6^A, N^6^-methyladenosine; mRNAs, messenger RNAs; miRNAs, micro RNAs; MSCs, mesenchymal stem cells; NRL, nucleotide recognition lid.
